# Community-Associated Methicillin-Resistant *Staphylococcus aureus*: An Enemy amidst Us

**DOI:** 10.1371/journal.ppat.1005837

**Published:** 2016-10-06

**Authors:** Eric F. Kong, Jennifer K. Johnson, Mary Ann Jabra-Rizk

**Affiliations:** 1 Graduate Program in Life Sciences, Molecular Microbiology and Immunology Program, University of Maryland, Baltimore, Maryland, United States of America; 2 Department of Microbiology and Immunology, School of Medicine, University of Maryland, Baltimore, Maryland, United States of America; 3 Department of Oncology and Diagnostic Sciences, Dental School, University of Maryland, Baltimore, Maryland, United States of America; 4 Department of Pathology, School of Medicine, University of Maryland, Baltimore, Maryland, United States of America; Tufts Univ School of Medicine, UNITED STATES

## Introduction

Methicillin-resistant *Staphylococcus aureus* (MRSA), or multidrug-resistant *S*. *aureus*, first reported in the early 1960s in the United Kingdom, are strains of *S*. *aureus* that through the process of natural selection developed resistance to all available penicillins and other β-lactam antimicrobial drugs [1]. Although the evolution of such resistance does not cause the organism to be more intrinsically virulent, resistance does make MRSA infections more difficult to treat and thus more dangerous, particularly in hospitalized patients and those with weakened immune systems [2]. MRSA can be spread from one person to another through casual contact or through contaminated objects, and a strain acquired in a hospital or health care setting is called health care–associated methicillin-resistant *S*. *aureus* (HA-MRSA) [2]. In fact, MRSA has become an important cause of nosocomial infections worldwide and is currently the most commonly identified antibiotic-resistant pathogen in United States hospitals [3–5].

However, although MRSA has been entrenched in hospital settings for several decades, it has undergone rapid evolutionary changes and epidemiologic expansion, spreading beyond the confines of health care facilities, where it is emerging anew as a dominant pathogen known as community associated-MRSA (CA-MRSA) [6]. The rapid dissemination of CA-MRSA strains among general populations in diverse communities has resulted in increasing reports of outbreaks worldwide [1]. In fact, in some regions, CA-MRSA isolates account for 75% of community-associated *S*. *aureus* infections in children, creating a public health crisis in the US [1,7]. In this article, we will provide a brief overview of what is known about the epidemiology and pathogenesis of community- associated MRSA and discuss how they differ from the strains originating in health care settings. Further, therapeutic and preventative measures available to combat the rising spread of this revamped pathogen are also discussed.

## Methicillin-Resistant *Staphylococcus aureus*

*S*. *aureus* is a major human pathogen that causes a wide variety of diseases, ranging from superficial skin and soft tissue infections to life-threatening conditions such as endocarditis, osteomyelitis, toxic shock syndrome (TSS) and infections associated with indwelling medical devices [4,8]. The asymptomatic carriage of *S*. *aureus* by humans is the primary natural reservoir, with the anterior nasal mucosa being the main ecological niche [9]. Colonization provides a reservoir from which the bacteria can be introduced when host defenses are breached, and therefore colonization increases the risk for subsequent infection [10]. Importantly, in addition to humans and domestic animals, livestock and fomites may also serve as adjunctive reservoirs, giving this bacterial pathogen dramatic relevance in veterinary medicine [11,12].

**Fig 1:**
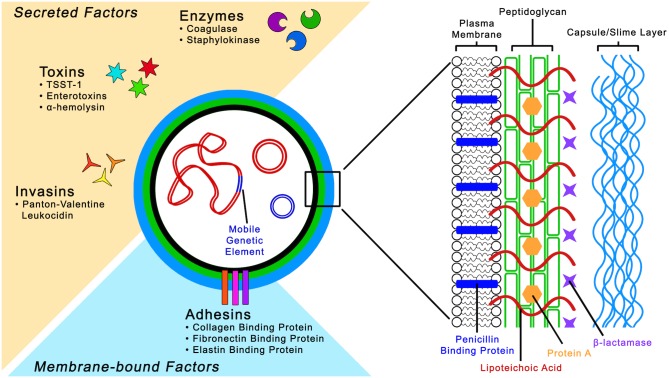
*Staphylococcus aureus* cell structure and pathogenic factors. *Staphylococcus aureus* has a complex cell wall structure composed of a thick peptidoglycan layer and polysaccharide capsule. In addition, *S*. *aureus* possesses an elaborate arsenal of structural and secreted virulence factors involved in toxin production, adherence to and invasion of host tissue, and immune evasion.

The virulence of *S*. *aureus* is multifactorial because of the combined action of an arsenal of virulence factors that facilitate tissue adhesion, immune evasion, and host cell injury [10]. These virulence determinants involve both structural factors, such as surface adhesins that mediate adherence to host tissues, and secreted factors, such as enzymes, which convert host tissue into nutrients (Fig 1). However, of more significance is the secretion of a variety of pyrogenic toxins known as superantigens; most notable are the Panton–Valentine leukocidin (PVL) and toxic shock syndrome toxin-1 (TSST-1) [13,14].

Importantly, the success of *S*. *aureus* as a pathogen has been attributed to the various measures it utilizes to protect itself from the host’s immune system. Among these strategies are production of complement inhibitory molecules, antibody-binding proteins, cytolytic peptides, pore-forming toxins, and most notably, production of polysaccharide capsules, which protect against phagocytosis [8,11,15–17]. Further, the species signature gene *spa*, which encodes protein A, also contributes to the prevention of opsonization and subsequent phagocytosis by binding to and neutralizing activity of the Fc region of immunoglobulin G (IgG). In addition, it also initiates a proinflammatory cascade in the airway by activating tumor necrosis factor receptor 1 (TNFR1) and B cells in concert with other ligands. Yet, despite what is known about the expansive armament available to this important bacterial pathogen, the role of different virulence factors in the development of staphylococcal infections remains poorly understood.

## What Is Community-Associated MRSA?

MRSA strains were once confined largely to hospitals, other health care environments, and patients frequenting these facilities; these health care–associated strains are known as hospital-associated MRSA (HA-MRSA) [1]. However, in a recent and dramatic evolutionary development, since the mid-1990s, there has been an explosion in the number of MRSA infections reported in the general populations [18]. This increase was associated with the recognition of new strains, which were named community-associated MRSA (CA-MRSA).

In 1999, following a report describing four pediatric fatalities in the mid-western US, CA-MRSA was recognized as a distinct clinical entity. Prior to that time, CA-MRSA cases were associated with intravenous drug users in Detroit, Michigan, and aboriginal populations in Western Australia. Beginning in 2000, CA-MRSA lineages were reported from numerous countries, with some lineages exhibiting restricted geographic ranges and others characterized by international epidemicity [1,19]. In 2000, the CDC created a case definition for MRSA infections occurring among healthy people in the community: any infection diagnosed in patients lacking health care–associated MRSA risk factors such as hospitalization, hemodialysis, surgery, presence of indwelling catheters, and other medical devices [1].

Infections with CA-MRSA typically occur in previously healthy individuals who likely have cuts or wounds and are in close contact with one another; therefore, outbreaks are characteristically reported in prisons, daycare centers, athletic teams, and schools [2,9]. In fact, CA-MRSA infections tend to occur in younger patients and are predominantly associated with skin and soft tissue infections and TSS. However, severe, life-threatening cases linked to several clinical syndromes, such as necrotizing pneumonia and necrotizing fasciitis, have been reported [7]. In contrast, HA-MRSA strains have been isolated largely from people who are exposed to the health care setting, where the patients are older and have one or more comorbid conditions, and these strains tend to cause pneumonia, bacteremia, and invasive infections. Although by definition, both CA-MRSA and HA-MRSA are resistant to all β-lactam antibiotics, important differences exist in epidemiology, microbiologic characteristics, clinical syndromes, and antimicrobial susceptibility patterns, indicating that these so-called “community-associated MRSA” have evolved independently of hospital MRSA [7].

## CA-MRSA Is Distinct from HA-MRSA Both Genetically and Phenotypically

**Fig 2:**
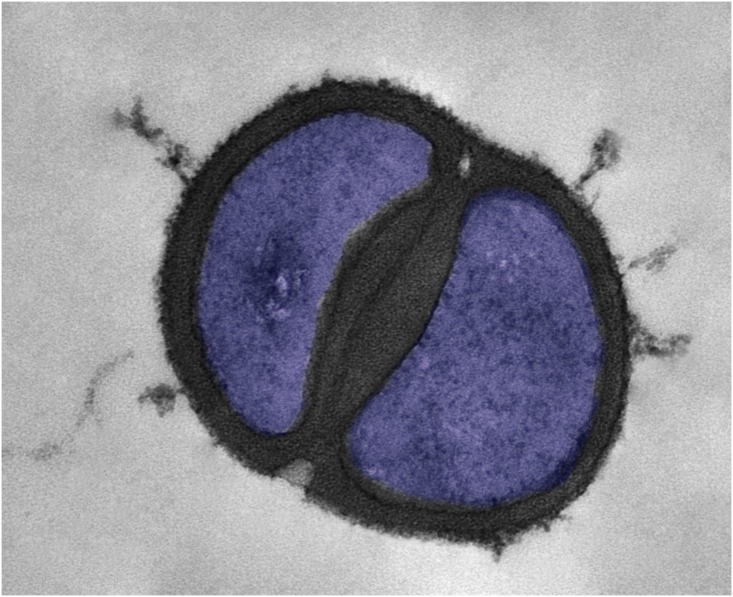
A false-colored transmission electron micrograph of USA300 strain *Staphylococcus aureus* cell.

CA-MRSA strains are now recognized as distinct clonal entities that differ from the traditional MRSA strains. In addition to the differences in epidemiological features, distinct clinical syndromes and antibiotic susceptibilities, the terms CA-MRSA and HA-MRSA have been used to call attention to genotypic differences [1]. Although the molecular determinants underlying the pathogenic success of CA-MRSA are not understood, studies have shown that the epidemic of CA-MRSA is caused by an extraordinarily infectious strain named USA300 (Fig 2) [9]. This strain, which originated in the community and is not related to strains from health care settings, is characterized by a phenotype of high virulence that is clearly distinct from other MRSA strains [18]. However, while USA300 (ST-8) in Europe was the first clone to be recognized, it is now clear that other clones with similar pathogenic properties dominate CA-MRSA isolates in other parts of the world [5,18].

CA-MRSA infections have mostly been associated with staphylococcal strains bearing the staphylococcal cassette chromosome mec type IV element and Panton–Valentine leukocidin genes. Methicillin resistance, signifying resistance to all β-lactam antibiotics, is mediated by the mecA gene encoding penicillin-binding protein 2a (PBP2a), which differs from other penicillin-binding proteins in that its active site does not bind methicillin or other β-lactam antibiotics [2]. Once acquired, the mecA gene is integrated into *S*. *aureus* chromosome and is thereafter contained within a genetic island called the Staphylococcal Cassette Chromosome mec (SCCmec). Although the presence of the SCCmec is common to nearly all MRSA strains, specific differences in the genetic island differentiate CA-MRSA from HA-MRSA. Whereas HA-MRSA strains carry a relatively large SCCmec, defined as types I–III, and are often resistant to many classes of non–β-lactam antimicrobials, CA-MRSA isolates carry smaller SCCmec elements, most commonly SCCmec type IV or type V. Further, CA-MRSA tend to be susceptible to narrow-spectrum non–β-lactams such as clindamycin, trimethoprim-sulfamethoxazole (TMP-SMX), and tetracyclines [1,20].

Another distinguishing genetic feature of CA-MRSA is that a high percentage of strains carry genes for Panton–Valentine leukocidin (PVL), which is largely absent from HA-MRSA strains. This exotoxin functions as a two-component pore-forming protein, encoded by the lukF-PV and lukS-PV genes, and acts as a leukocidin that can lyse the cell membranes of human neutrophils [2,7,16]. Therefore, PVL is hypothesized to be responsible for the enhanced pathogenicity of CA-MRSA strains. The first clinical isolate known to carry the PVL genes in the CA-MRSA era was reported in 2003, and approximately 60% to 100% of CA-MRSA strains have been shown to carry these genes, which can spread from strain to strain by bacteriophages [1]. However, although PVL has been closely linked to infections caused by CA-MRSA strains and shown to be instrumental in producing necrotic skin lesions and necrotizing pneumonia, it is not known with certainty how this toxin contributes to their fitness and/or virulence.

Although it has been speculated that determinants such as PVL encoded on mobile genetic elements (MGEs) have a predominant impact on virulence, recent reports seem to imply that the contribution of these MGEs to CA-MRSA virulence may be comparatively minor. In fact, based on genome comparisons and epidemiological data, findings from one study indicated that high expression of core genome-encoded virulence determinants—such as the global virulence and quorum-sensing regulator *agr*—rather than the acquisition of additional virulence genes, may have a more profound impact on the evolution of virulence [18]. However, PVL was proposed to have an important role in defining the virulence gene expression pattern, which results in the increased virulence potential [18].

## Treatment, Prevention, and Future Perspectives

Treatment options for CA-MRSA include incision and drainage, oral or parenteral antibiotics, and topical therapy. However, there are relatively few antibiotic agents available to treat MRSA, as the worldwide spread of multidrug-resistant clones during the past several decades has severely limited treatment options [20]. The glycopeptide antibiotic vancomycin is one of the few antibiotics that remains effective against MRSA [21]. However, with the antibiotic pressure exerted by the increasing use of vancomycin to treat MRSA infections, in 2002, the first clinical isolate with high-level vancomycin resistance, vancomycin-resistant *S*. *aureus* (VRSA), was reported in the US [22]. However, these strains are rare and there is little evidence for increasing frequency. The VRSA strains carry transposon Tn*1546*, acquired from vancomycin-resistant *Enterococcus faecalis*, which is known to alter cell wall structure and metabolism [21]. Therefore, clinical reliance on vancomycin may no longer be possible [1]. The emergence of vancomycin-intermediate *Staphylococcus aureus* (VISA), which were first identified in 1996 and have since been detected globally, has further compounded the therapeutic challenges. Although the resistance mechanism of these strains with reduced susceptibility to vancomycin is not fully clear, it was predominately associated with cell wall thickening and vancomycin binding, thereby restricting access of the drug to its site of activity [23,24]. Clindamycin is an excellent oral option for the treatment of CA-MRSA, as in addition to its efficacy it also has the benefit of inhibiting toxin production and therefore has a theoretical benefit in patients with toxic shock or other toxin-mediated complications [20]. The limitations of the available agents combined with the slow rate of development of new antibiotic classes have raised the notional possibility of untreatable multidrug-resistant *S*. *aureus* infections [1]. Therefore, continuous efforts should be made to prevent the spread and the emergence of resistance by early detection of the resistant strains [25].

Often, outbreaks of CA-MRSA have been in populations in which close contact appears to be the common characteristic. Although data on the effectiveness of strategies to prevent new and recurrent CA-MRSA infections are currently limited, hygiene, environmental cleaning, and proper wound care are essential components to infection control [2]. However, attempts to contain MRSA using current infection control based in health care facilities are unlikely to succeed without a similar effort to control spread in the community. Until these studies are conducted, health care practitioners will need to extrapolate from infection control guidelines for controlling MRSA within the hospital.

The increasing burden of CA-MRSA underscores the need to find innovative therapeutics for MRSA disease. Although CA-MRSA isolates are typically susceptible to many non–β-lactam antibiotics, there is recent emergence of multidrug-resistant CA-MRSA, thus confounding the current serious public health problem [18]. An effective multicomponent vaccine may be the only effective long-term solution against the spread of CA-MRSA [16]. The role of capsules as an important immune evasion mechanism supports the inclusion of capsular polysaccharides in the formulation of prophylactic vaccines [17]. Further, secreted products, such as the staphylococcal protein A (SpA), may also be exploited for the development of vaccines and therapeutics [15]. Thus, comprehensive understanding of the pathogen’s ability to manipulate the host immune response is crucial for the development of efficacious vaccines against CA-MRSA [8,11].

CA-MRSA infections have become commonplace, and their worldwide emergence in healthy individuals represents an ominous threat. Ironically, CA-MRSA strains are now being introduced from their site of origin in the community into the hospital, reversing the epidemiologic cycle. In fact, in some hospitals, CA-MRSA are displacing classic health care–associated strains of *S*. *aureus*, supporting the hypothesis that CA-MRSA may be more fit [26]. Mathematical modeling demonstrates difficulty in the epidemiologic control of CA-MRSA in the face of its increased prevalence in the community and the increasingly daunting tasks for infection control programs. There is an acute need to reduce the global burden of infections, and therefore, as the definitions of a “community-associated” infection continue to evolve, it is imperative that studies are directed toward examining effective prevention and outbreak control strategies. Importantly, increased vigilance in the diagnosis and management of suspected and confirmed staphylococcal infections is warranted [7].
